# 

**Published:** 1922-04

**Authors:** 


### ANNOUNCEMENTS

### Colorado State Board of Dental Examiners

The next meeting of the Colorado State Board of Dental Examiners will be at Denver Colorado, June 6, 1922. For further information address Wm. H. Flint, Secretary, Littleton, Colorado.

### Massachusetts Dental Society

The fifty-eighth annual meeting will be held in Worcester, Massachusetts, at Mechanics Hall, 321 Main Street, May 2, 3 and 4, 1922. A cordial invitation is extended to all members of recognized dental societies. W. Vernon Ryder, Secretary, 175 Newbury St., Boston, Massachusetts.

Thanking you in advance, I am W. Vernon Ryder, Secretary.

### May Meeting

Illinois, at Springfield, May 9th to 11th. Indiana, at Indianapolis, May 15th to 18th. New York, at Rochester, May 11th to 13th. Tennessee, at Memphis, May 8th to 11th. Northern Ohio meeting, June 5th-7th.

### Communication

Editor "The Dental Register."

I was very much interested in your article in the February number on "Occupational Dermatitis." But it occurs to me that there are better ways of avoiding it than those suggested.

I have been developing films for three years and use Eastman's X-ray Developing powders. Of course they contain metol and when I first began to use them I immersed my middle finger of the right hand into the solution for each film. I have a rather resistant skin and wasn't bothered much till I had a rush week. Then I had the full benefit of the trouble. It didn't last long for me as I soon decided that the developer was to blame.

I use a blind cabinet. The Eastman film packet and an old style folded Eastman film packet which is empty and a Dennison clip are fastened together by slipping the clip over the two packets. Then after the door is closed, the clip and empty packet are placed between the last two fingers of the left hand and held there while the film packet is being opened. When the pair of films are removed one is placed on the clip and then the clip is laid in the left sleeve while the other one is placed in the empty film pack for future possible need. Three films are developed at one time, the solution being in small wine glasses. My fingers don't touch the solution because it is not necessary and I am careful.

If you, doctor-reader, have a good many films to develop each day and this method is not rapid enough for you you should ask enough for your work that you can afford to have the proper equipment to carry on the work. For medium large amounts of the work, portable dark rooms with ruby glass windows may be had that will enable quite an increase in speed. For large practices a good dark room is best.

Keeping your fingers out of the developer is the ounce of prevention that is worth a good deal more than wearing rubber gloves, finger cotes, acid stop baths and is a whole million times more manly than letting the assistant do it with her fingers in the solution. If your assistant gets metol poisoning, it is your own fault, doctor, and you should be made to *pay* the damages, not the accident insurance companies!—Dr. H. E. Jones, Portland, Indiana.

[We have submitted this communication to the Radiographic department of the University of Michigan and find that Dr. Jones is quite right; and that no one should purposely place his hands in the developer.—*Editor* REGISTER.]

